# Implementation models and frameworks used to guide community-based physical activity programs for children: a scoping review

**DOI:** 10.1186/s12889-023-16465-2

**Published:** 2023-08-23

**Authors:** Emma Ostermeier, Shauna M. Burke, Jason Gilliland, Patricia Tucker

**Affiliations:** 1 Health and Rehabilitation Sciences, Faculty of Health Sciences, Western University, London, ON Canada; 2 School of Health Studies, Western University, London, ON Canada; 3 Children’s Health Research Institute, London, ON Canada; 4 Human Environments Analysis Laboratory, Department of Geography and Environment, Western University, London, ON Canada; 5 Department of Geography & Environment, Western University, London, ON Canada; 6 Department of Epidemiology & Biostatistics, Western University, London, ON Canada; 7 Department of Paediatrics, Western University, London, ON Canada; 8 Lawson Health Research Institute, London, ON Canada; 9 School of Occupational Therapy, Faculty of Health Sciences, Elborn College, Western University, 1201 Western Rd, ON N6G 1H1 London, Canada

**Keywords:** Population-level, Intervention, Dissemination, Development, Youth

## Abstract

**Background:**

The implementation of community-based programs is key to effective, sustainable initiatives that can support population-level changes in children’s physical activity. The purpose of this scoping review was to explore the implementation models and frameworks used to develop (process models), explore (determinant frameworks), and/or evaluate (evaluation frameworks) community-based physical activity programs for children. Also, the foundational components of the implementation models and frameworks and practical application in real-world settings were described.

**Methods:**

The methodological framework developed by Arksey and O’Malley (2005) and the updated recommendations from Levac, Colquhoun and O’Brien (2010) were used to search, identify, and summarize applicable studies. This review also met the requirements in the Preferred Reporting Items for Systematic Reviews and Meta-Analyses Scoping Reviews Checklist (PRISMA-ScR). A detailed search of six databases and three academic journals was conducted. Information about the article, the program, and the implementation model/framework were extracted and summarized.

**Results:**

The search retrieved 42,202 articles, of which 27 met the inclusion criteria. Eleven process models, one determinant framework, and two evaluation frameworks were identified. Nineteen components were developed from the models and frameworks. Tailoring, situational analysis, and element identification were common components among the identified models and frameworks.

**Conclusions:**

Since the execution of interventions is vital for creating successful health-promoting initiatives, researchers and program developers should consider using implementation models and frameworks to guide their community-based physical activity programs. Further research examining the application of new and existing implementation models and frameworks in developing, exploring, and evaluating community-level programs is warranted.

**Supplementary Information:**

The online version contains supplementary material available at 10.1186/s12889-023-16465-2.

## Background

A multi-level approach to behaviour change is a recommended strategy in health promotion as it considers individual, societal, and environmental health determinants [1]. Community-based public health initiatives can utilize a multi-level approach to identify the factors that influence health behaviours and integrate findings into the program structure to improve the targeted outcomes [2]. Community-based programs have become a prominent strategy for engaging children in the World Health Organization’s recommended 60 min of daily moderate-to-vigorous physical activity (MVPA) per day [3]; however, programs need to account for a diverse group of determinants that positively (e.g., parental support, interest in activities that elicit MVPA, local recreation spaces) or negatively (e.g., lack of local recreation spaces, insufficient transportation options, and financial constraints) shape children’s physical activity participation [4–6]. Encouraging children to engage in greater amounts of physical activity has been a priority for the World Health Organization [7] due to the beneficial effects on health and well-being, including cardiometabolic health [8], bone mineral density [8], anxiety [9], depression [9], academic achievement [10], and cognitive functioning [11]. Considering that extracurricular activities, such as sports and organized programming, are popular forms of physical activity for children, providing accessible after-school and weekend offerings can be a valuable approach to improving children’s physical activity levels [12].

Unlike clinical settings, community settings are unpredictable environments where researchers and program providers cannot control for all confounders and societal conditions; consequently, programs, when scaled-up from clinical to community settings, are not always effective [13, 14]. To create beneficial changes in children’s physical activity, program developers need to design appropriate and feasible programs and they need to ensure they are implemented as intended. This requires researchers, program developers, and program providers to: (1) establish evidence-based, tailored plans when implementing community-based programs; (2) evaluate the adoption and delivery of the program by the providers and participants; and (3) identify any necessary adjustments that will create an effective program structure that can be implemented in a community setting [15–18]. If aspects of the program are missing or are not implemented as intended, the findings can result in misleading conclusions about program effectiveness [16].

The field of implementation science was developed to better support the translation of evidence-based interventions into community settings [19]. Specifically, strategies such as implementation models or frameworks are one approach researchers and program developers can employ to guide community-based physical activity programs. Unlike classic theories derived from other disciplines, implementation models and frameworks are developed in the field of implementation science to identify the factors that influence program outcomes and support the use of knowledge in practice [20]. Implementation models and frameworks are made up of a variety of components that act as a foundation for an evidence-based process for executing community-based programs [21]. They can also help researchers assess implementation outcomes. Unlike service (e.g., efficiency and equity) and client outcomes (e.g., health behaviours and satisfaction), implementation outcomes consist of the actions used to implement new programs and practices, such as fidelity, acceptability, costs, and sustainability [22]. Ultimately, using implementation models and frameworks to develop, explore, and evaluate community-based programs can support the creation of feasible physical activity interventions that can be effectively administered by participating stakeholders after researchers have left the program [23]. Three types of implementation models and frameworks are the focus of this review: (1) process models help develop programs by describing the translation of research into practice, (2) determinant frameworks explore programs to understand the factors that influence implementation outcomes, and (3) evaluation frameworks examine the implementation of programs [21].

While previous reviews have examined the relationship between program implementation and children’s physical activity outcomes, the types of models used, and the factors that influence implementation, have largely focused on school settings [24, 25]. Schools are an advantageous location for physical activity initiatives targeting children as the curriculum contains designated activity times and children are readily available [26]. Community-based programs face additional social (e.g., parental support) and environmental (e.g., neighbourhood safety) challenges; as a result, the findings from school-based interventions may not be generalizable to a community setting. Thus, a review focusing on the implementation of community-based programs targeting children’s physical activity is needed.

The aim of this scoping review was to explore the implementation models and frameworks used to develop, explore, and/or evaluate community-based physical activity programs for children ages 5 to 12 years. The primary objective of this study was to identify the models and frameworks employed by researchers and program developers to support the implementation of community-based physical activity programs for children. As a secondary objective, the key components of the models and frameworks, and how the models and frameworks have been used in practice are highlighted.

## Methods

### Study design

A scoping review was deemed appropriate as this study aimed to explore the breadth of the literature on the role of implementation models and frameworks in community-based physical activity programs [27]. The review followed the five stages outlined in Arksey and O’Malley’s [28] scoping review methodological framework and integrated the updated recommendations by Levac, Colquhoun, and O’Brien [29]. This review also met the standards in the Preferred Reporting Items for Systematic Reviews and Meta-Analyses extension for Scoping Reviews Checklist (PRISMA-ScR; see Table S1) [30].

### Stage 1: Identifying a research question

The aim of this scoping review was to explore the implementation models and frameworks used for community-based physical activity programs for children. To meet this aim, we created three research questions:

- What models and frameworks have been used to support the implementation of community-based physical activity programs for children?
- What were the key components that form the models and frameworks?
- How were the models and frameworks used in practice?

### Stage 2: Identifying relevant studies

Following an initial consultation with a systematic reviews librarian, six electronic databases were searched for relevant articles: CENTRAL, MEDLINE (Ovid), Embase (Ovid), Scopus, CINHAL, and Web of Science. The search string included a variety of terms related to physical activity AND children AND implementation science OR implementation frameworks and models AND community-based programs (see Table S2). ﻿Truncation symbols were used to account for variations in the search terms and to increase the sensitivity of the search. The search was originally run in December 2021 and was re-run in March 2022 to ensure all published articles were captured. Relevant articles were also identified via journal searches in *Implementation Science*, the *Journal of Translational Behavioral Medicine*, and the *International Journal of Behavioural Nutrition and Physical Activity*. The implementation model and framework references cited in the included articles were also extracted and reviewed.

### Stage 3: Study selection

Search results were imported into Covidence screening and data extraction software. Duplicate citations were automatically removed by the software. ﻿Titles and abstracts were screened by two members of the research team. Subsequently, the full-text screening was carried out independently by two researchers. Disagreements were resolved by an alternative member of the research team.

#### ﻿Eligibility criteria

All peer-reviewed publications that used a model or framework to support the implementation of a community-based physical activity program targeting children were considered for inclusion. A program was defined as any intervention or initiative with planned activities and intended outcomes that were developed and executed by a research team or organization. To be eligible for inclusion in this scoping review, articles had to meet the following criteria: (a) the target population was children between the ages of 5 to 12 years, (b) the program had to transpire in a community setting (e.g., before-school, after-school or weekend programs), (c) physical activity was the primary target health behaviour and/or outcome, (d) the article explicitly referred to an implementation model or framework that guided the development, exploration, or evaluation of their program, and (e) full-text version of the article was available in English. Articles were excluded if the mean participant age was above or below the age range or if the program was home-, school-, web-, or multi-setting-based (e.g., school and community settings). High school-aged children (13–18 years) were excluded from the target population as children’s physical activity preferences alter during the transition from childhood to adolescence, potentially due to biological changes or new social pressures and expectations [31]; consequently, strategies for promoting physical activity among older cohorts may differ from those utilized for elementary school children [32]. Theories were excluded from this review as they provide a more general explanation of concepts and the relationships that lead to an outcome [21]. In implementation science, the terms ‘theory’, ‘model’ and ‘framework’ tend to be used interchangeably [33]; however, they each have distinct purposes. Models and frameworks were selected for this review as they are descriptive, with models offering a defined depiction of one aspect that leads to the outcome of interest and frameworks outlining concepts that are believed to result in a specific outcome [21].

### Stage 4: Charting data

The data were extracted from the articles and entered into an Excel table developed by the research team. An initial extraction of three articles was conducted by a member of the research team to ensure the table was comprehensive and captured all important details from the studies. Extracted data included journal details, study characteristics, setting, sample population, program description, and implementation model or framework details.

### ﻿Stage 5: Collating, summarizing and reporting the results

The frequency of the study characteristics and the identified models and frameworks were reported in a descriptive numerical summary of the included articles. The categories for the models and frameworks were guided by Nielsen’s [21] definitions for implementation theories, models, and frameworks. The identified models and frameworks were classified as a process model, determinant framework or evaluation framework based on the description provided in the referenced model/framework manuscript and their application in the identified article(s). To further understand the identified models and frameworks, the components were explored using an inductive content analysis [34]. A content analysis has been described as an appropriate approach for scoping reviews when examining key characteristics or informing frameworks [35] and allowed the research team to get more depth from the data on the components that are consistently or infrequently integrated into implementation models and frameworks [29]. Each implementation model and framework was reviewed and its key features and/or different phases were coded. Codes were grouped into categories to develop the components, which were further grouped into overarching topics. Subsequently, a narrative summary describing how researchers and program developers applied the models and frameworks in practice was conducted. The findings were then used to identify gaps in the literature and to develop preliminary recommendations for researchers and program developers on which models and frameworks have been used to implement community-based physical activity programs.

## Results

**Fig. 1:**
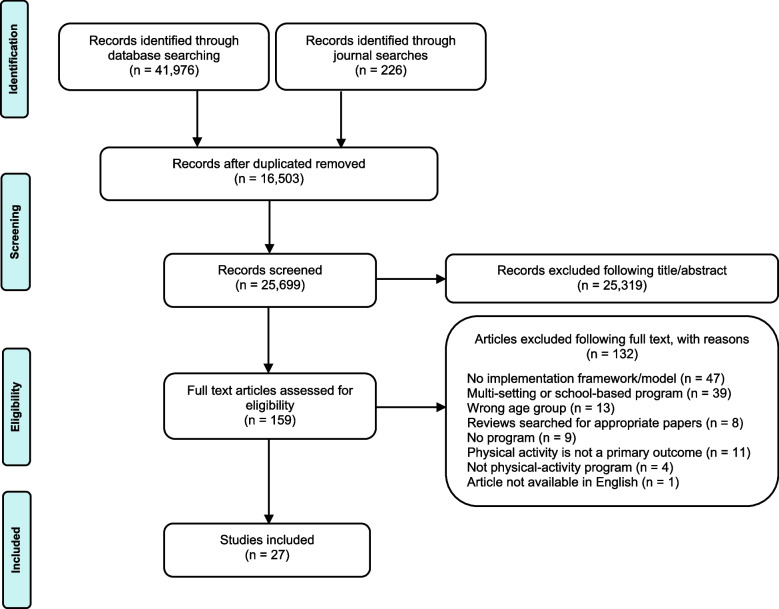
PRISMA diagram of the database search and screening process

The search yielded 42,202 articles; following the removal of duplicates, 25,699 unique articles remained. Title and abstract screening removed 25,319 articles. A total of 159 articles were retrieved and underwent full-text screening. Overall, the screening process resulted in 27 articles that met the eligibility criteria of this review. The PRISMA diagram for the literature search is reported in Fig. 1; a full description of the included articles is provided in Table S3.

### Study characteristics

The included articles were recent in publication, ranging from 2010 to 2021. The 27 articles in this review consisted of 12 case studies [21, 36–46], 4 study protocol papers [47–50], 3 randomized control trials [51–53], 2 quasi-experimental trials [54, 55], 2 longitudinal studies [56, 57], 1 prospective evaluation [58], 1 cross-sectional study [59], 1 review [60], and 1 systematic review [61]. A majority of the articles used mixed methods (e.g., surveys, interviews, workshops, community reports) to report on the implementation of their programs (*n* = 13) [21, 38–43, 47, 51, 52, 57, 58, 60], while the remained focused on quantitative (*n* = 10) [43, 45, 48–50, 53–55, 59, 61] or qualitative measures (*n* = 4) [36, 42, 44, 56].

The studies took place in the USA (*n* = 15) [21, 36, 39, 40, 43, 44, 46, 48–50, 53–55, 57, 60], Canada (*n* = 6) [37, 41, 42, 56, 58, 59], United Kingdom (*n* = 4) [38, 41, 45, 46], Australia (*n* = 1) [39], and one article used data from the USA, Canada and Australia [55]. They were primarily conducted in urban spaces (*n* = 8) [36, 40, 42, 48, 54, 56, 58, 60], with the remaining taking place in rural and/or remote areas (*n* = 6) [21, 37, 41, 43, 44, 50]; urban, suburban, and rural communities (*n* = 2) [39, 53]; or not specified environments (*n* = 10) [38, 45, 46, 49, 51, 52, 55, 57, 59, 61].

### Implementation models and frameworks

In the 27 included articles, 14 models and frameworks were identified (Table 1). Similar to the findings from previous reviews examining community-based physical activity interventions for children [16, 17, 62], many studies omitted an implementation model or framework during program development, exploration, or evaluation. This was the most frequent reason why articles were excluded during the full-text screening (*n* = 47, 36%). Alternatively, many programs opted to use classic theories to guide their programs, such as the self-determination theory [63], social cognitive theory [63], theory of planned behaviour [64], the transtheoretical model [65], socio-ecological model [66, 67], and theory of triadic influence [68].

**Table 1:** Description of the process models, determinant frameworks, and evaluation frameworks used in community-based physical activity programs for children

Framework^A^	# of Articles	Components	Application	Data Collection Techniques^B^
**Process Models (*n* = 11)**				
Analysis Grid for Environments Linked to Obesity (ANGELO) Framework (Swinburn, Egger & Raza, 1999)	2	• Situational Analysis • Partners and Motives • Element Identification	To ﻿build on the local context by identifying key aspects of their community that influence health behaviours and focused on activities determined to be important and achievable by the community	• Community Reports • Surveys • Workshops
Behaviour Change Wheel (Michie, van Stralen & West, 2011)	3	• Change Agents • Element Identification	To adapt the current program structure to meet the needs of the community or a specific sub-population	• Observations • Interviews • Questionnaires
Community-based Prevention Marketing (CBPM) Framework (Bryant et al., 2007)	1	• Community Participation • Element Identification • Program Management • Tailoring • Situational Analysis • Partner Organization	To design a multi-media campaign to engage children in physical activity	• Workshops • Surveys • Focus Groups • Interviews
Foster-Fishman et al.'s (2007) Systems Framework (Foster-Fishman, Nowell & Yang, 2007)	1	• Community Partnership • Situational Analysis • Change Agents • System Interactions • Element Identification • Partner Organization	To facilitate collaborations with institutional and community stakeholders in the development and evaluation of the program	• Observational Tools (e.g., SOSPAN and SOPLAY)
Integrated Capacity Building Framework (Foster-Fishman et al., 2001)	1	• Partnership Management • Partner Organization • Situational Analysis • Tailoring	To build capacity for ﻿policy, systems, and environmental changes to engage children in healthy behaviours	• Surveys • Interviews
Knowledge-to-Action (KTA) Framework (Graham et al., 2006)	1	• Situational Analysis • Tailoring • Program Management • Knowledge Creation • Element Identification	To ﻿guide the development and implementation of the program	• Workshops
Life Needs Model (King et al., 2002)	1	• Equity and Accessibility • Tailoring • Partners and Motives • Element Identification	To consider the various levels of determinants to find appropriate program strategies that support healthy behaviours	• Questionnaires • Focus Groups
Management Model for Sport and Physical Activity Community-based Partnerships (Parent & Harvey, 2009)	2	• Partners and Motives • Tailoring • Knowledge Creation • Partnership Management • Partner Organization	To identify the critical factors that result in successful partnerships that help inform programs	• Interviews
Social Marketing Model (Lee & Kotler, 2016)	1	• Recruitment • Element Identification • Tailoring • Program Management	To guide the development of a multi-media campaign that targets children's physical activity	• Ethnographic Research • Focus Groups • Interviews • Telephone Surveys
Strategies To Enhance Practice—Physical Activity (STEPs-PA) (Collins, Murphy & Bierman, 2004)	2	• Fidelity • Training • Equity and Accessibility • Tailoring	To evaluate the implementation and design of the program	• Program Documents (e.g., schedules) • Observational Tools (e.g., SOSPAN) • Objective or Subjective Physical Activity Measures
Typology of Cultural Adaptation and Programme Theory of Adapted Health Promotion Interventions (Liu et al., 2012)	2	• Tailoring • Recruitment • System Interactions • Program Management • Equity and Accessibility	﻿To ensure that appropriate cultural adaptations are made to all aspects of the tailored version of the program to ensure they meet the needs of the community	• Interviews • Observations
**Determinant Frameworks (*n* = 1)**				
A + quality improvement toolkit (Wiecha, Hannon & Meyer, 2013)	1	• Tailoring • Program Management • Program Design • Maintenance	To improve the implementation of the program	• Interviews • Focus Groups
**Evaluation Frameworks (*n* = 2)**				
Hybrid Type 3 Evaluation Design (Curran et al., 2012)	1	• Partnership Management • Efficacy/Effectiveness • Fidelity • Program Management • Change Agents	To design a model that enables researchers to evaluate implementation strategies	• Feedback Survey • Program Documents (e.g., attendance logs), • Fidelity • Questionnaire • Interviews
RE-AIM (Glasgow, Vogt & Boles, 1999)	11	• Reach • Efficacy/Effectiveness • Adoption • Fidelity • Maintenance	To evaluate the dissemination quality and effectiveness of a program	• Questionnaires • Surveys • Observations (e.g., staff check-ins) • Program Documents (e.g., attendance logs) • Objective or subjective physical activity measures

^A^Models and frameworks were classified based on the reference manuscript and how they were used in the study

^B^The data collection strategies used in the included articles

*SOSPAN:* System for Observing Staff Promotion of Activity and Nutrition, *SOPLAY:* System for Observing Play and Leisure Activity in Youth

Eleven of the 14 identified models and frameworks were classified as process models. Compared to determination and evaluation frameworks, process models were the most diverse implementation model/framework type. Specifically, the models differed based on the subject matter and the number of implementation components included. In some cases, the model specialized in a specific area of program development. For instance, Foster-Fishman et al.’s [69] systems framework and Parent and Harvey’s [70] management model for sport and physical activity community-based partnerships focused on integrating community members and organizations into the development of community-based programs, while the Analysis Grid for Environments Linked to Obesity (ANGELO) framework [71] integrates community stakeholders and organizations into the planning process in order to understand and incorporate the local context into the program design. Alternatively, process models can be comprehensive by including a variety of aspects that support program development, such as the Community-Based Prevention Marketing (CBPM) framework that includes program recruitment, tailoring, and management of participating organizations and the program in general [72]. Across the 27 articles, the Behaviour Change Wheel was the most commonly employed process model (*n* = 3); it was used by researchers to either tailor programs for underserved communities [47, 52] or to select strategies that can support changes to children’s physical activity [51].

Only one determinant framework was identified from the search. The A + quality improvement toolkit is a checklist of items that can be used to assess the knowledge, execution, and resources to improve a program’s capacity to make health behaviour changes [36]. In practice, Wiecha, Hannon, and Meyer [36] examined the application of this framework by conducting interviews and focus groups with program directors and participating organizations to determine if the toolkit helped improve the implementation of the YMCA afterschool programs.

Finally, two evaluation frameworks were found in the search. RE-AIM was the most prevalent framework for evaluating the implementation of programs (*n* = 11) [21, 38, 41, 44, 45, 48, 50, 54, 57, 59, 61]. The RE-AIM framework was used to assess the outcomes of programs [21, 38, 41, 54, 61], evaluate the quality of the program dissemination [50, 57], and examine the transition of a program into a community setting [44, 45, 48, 59]. In two cases, the RE-AIM framework was also used to guide the development and evaluation of the ﻿EPIC Kids study [48] and the Children’s Healthy Living program [44], showing the diversity of the framework. The remaining study used the ﻿Hybrid Type 3 Evaluation Design to evaluate the scale-up and implementation of the ﻿Mind, Exercise, Nutrition … Do it! Intervention [58].

### Model and framework components

A total of 19 components were identified across the 14 models and frameworks. The definitions of the components developed by the research team are provided in Table 2. The components covered a variety of topics, including adapting programs to the local context, building capacity and partnerships, uptake of the program by service providers and families, quality of the program delivery, development of the program structure, and assessment of program implementation.

**Table 2:** Definitions of components (*n* = 19) used across the three types of implementation models and frameworks

Topic	Component	# of models/frameworks (*n* = 14)	Definition
Adapting programs to the local context	Situational Analysis	5	Examining the community you intend to implement the program in, including demographics, health data, and social and physical environments (e.g., to find barriers, facilitators, etc.)
	Tailoring	9	Altering programs to the local context to recognize that there is a need to offer different resources and strategies to meet the needs of the community based on the political, demographic, economic, and socio-cultural context
Building capacity and partnerships	Community Partnership	2	The integration of community members and researchers as equal partners in every phase of the project
	Partner Organization	4	The formalized processes and procedures for partnership, such as the quantity and quality of the communication among partners and decision-making processes
	Partners and Motives	3	Considering the types of people and organizations that fit the goals of the project, and understanding their reasons for joining the partnership and what they will contribute to the project
	Partnership Management	3	Co-ordination of participating organizations, including the collaboration amongst partners, capacity, accountability, evaluation of partnership, and commitment to implement the program
Uptake of the program by service providers and families	Adoption	1	The proportion of the settings and staff that participated in the program
	Equity and Accessibility	3	Program engagement and activity offerings appeal to various groups in the community and can be utilized by the target audience
	Reach	1	The proportion of the target population (e.g., patient or employee) that participated in the program
	Recruitment	2	Identifying whom you want to participate in the program and how will you encourage them to partake
Quality of the program delivery	Program Management	4	The management of the program, including the feasibility, implementation, evaluation, and maintenance
	Training	1	Professional development training targeting program leaders and frontline staff to produce high-quality daily offerings of physical activity
Development of the program structure	Change Agents	3	Identifying the norms, resources, regulations, and decision-making processes that cause, maintain, and change the health behaviour
	Element Identification	7	Factors essential for program effectiveness (e.g., schedules, budget) that must exist for basic program delivery
	Knowledge Application	2	The development of the project is guided by literature on previous programs
	System Interactions	2	How different features of the program interact to positively or negatively affect health behaviours
Assessment of program implementation	Efficacy/Effectiveness	2	Success rate (if implemented as intended); this was determined by the positive outcomes minus negative outcomes. Also examining physiological, behavioural, quality of life, and participant satisfaction outcomes
	Fidelity	3	The extent to which the program is implemented as intended in the real world
	Maintenance	2	The extent to which the implementation of the program and the behaviour changes are sustained over time

Tailoring was the most common component and it appeared in a majority of the process models and determinant frameworks (*n* = 9) [36, 70, 72–78]. Authors described tailoring as an important aspect of program implementation as it can help engage a specific group in physical activity [78] or it can address a community’s capacity to increase physical activity among children [70]. To appropriately tailor programs to the community during the development stage of the program, five of the seven process models that included tailoring also integrated a situational analysis. Through ethnographic research [60] and interviews with community members and organizations [56], researchers and program developers gained information on the demographic, social, and environmental organization of the community, ultimately adapting the program to ensure that it meets the unique needs of the community.

While tailoring programs to the community context was common in the identified models and frameworks, the uptake of the program and considerations of whom the program is recruiting were not commonly included. Defining the target population (i.e., recruitment) was specified in the ﻿Social Marketing Model [76], while the equity and accessibility of community-based physical activity programs were included in the Life Needs Model [75] and STEPs-PA framework [49, 77]. Only one model, the Typology of Cultural Adaptation and Programme Theory of Adapted Health Promotion Interventions, incorporated both a defined target population and an assessment of the program’s accessibility [78]. The RE-AIM framework does include the concept of program uptake, examining the participation in the program by program staff (i.e., adoption) and by the target population (i.e., reach) [13]. Integrating these components through interviews with community stakeholders and families [38, 47], researchers and program developers can incorporate the advised recruitment strategies into the program’s design, which can help engage a diverse group of program participants.

Additionally, not many models or frameworks suggested an examination of the previous literature (Knowledge Application; *n* = 2). The Knowledge-to-Action (KTA) Framework emphasizes the importance of using research and experiences to guide the development and evaluation of programs as reviews of the literature and workshops are a valuable way to integrate the diverse perspectives of different community groups and organizations into physical activity programs [69]. For instance, in practice, Wurz et al. [42] used the KTA framework to develop and implement the Bounce Back League, a sports program for trauma-sensitive children. The authors used the knowledge of the trauma-sensitive sports practices within the Boys and Girls Club and involved experts in the field of trauma, sport, and program evaluation during program development to create a sustainable, scalable intervention [42].

Another component that frequently appeared in process models was element identification (*n* = 7) [69, 71, 72, 74–76, 79]. This component is part of developing effective community-based programs as it identifies the essential features in the program structure that must be met in order to address children’s physical activity behaviours. While the identification of individual components was prominent, systems interactions (how the relationships between the different aspects of the program structure interact to influence health behaviours positively or negatively) were only examined in Foster-Fishman et al.’s [69] Systems Framework and the Typology of Cultural Adaptation and Programme Theory of Adapted Health Promotion Interventions [78].

The topic of building capacity and partnerships in the community was also common among process models. This topic consisted of the components focused on the formalized processes and procedures for partnership (partner organization; *n* = 4) [68–70, 72], the types of organizations that are a good fit for the program and the organizations’ reasons for participating in the program (partners and motives; *n* = 3) [70, 71, 75], the administration of the program (partnership management; *n* = 3) [70, 72, 80], and the integration of community members and researchers into the development of the program (community partnership; *n* = 2) [69, 72].

When evaluating program implementation with an evaluation framework, all of the evaluation frameworks focused on the short-term outcomes of the program during their implementation evaluation via effectiveness/efficacy and fidelity [13, 49, 69]. While the component of effectiveness/efficacy examines the health behaviour outcomes that are targeted by the program via objective (e.g., accelerometers) [48, 49] or subjective measures (e.g., questionnaires) [57, 59], fidelity assesses the extent to which the program plan is implemented as intended [38, 41]. By selecting a model or framework that integrates both components, researchers can determine if the program was implemented as intended and, if so, was the program able to make health behaviour changes. Alternatively, the concept of maintenance or the long-term effects of the program were only considered in the RE-AIM framework [13] and the A + quality improvement toolkit [36].

## Discussion

The purpose of this scoping review was to examine the models and frameworks used to implement community-based physical activity programs for children. Specifically, the models and frameworks employed in the literature to develop, explore, and/or evaluate the implementation of community-based physical activity programs for children were reviewed, and the key components of the model or frameworks and how they were used to guide these community-based physical activity programs were highlighted. A number of findings warrant discussion.

Similar to the findings from previous reviews [16, 17, 62], many studies examining community-based physical activity programs did not report an implementation model or framework. The findings from this review indicate that researchers tend to use classic theories to guide their interventions instead of an implementation model or framework. While theories related to decision-making processes, social networks and community organizations can help explain the mechanisms of change in implementation, models and frameworks have practical application advantages by offering a guide containing the aspects that can lead to successful program implementation [81]. As implementation aims to translate knowledge into practice to create evidence-based interventions, using a model or framework can improve the quality of community-based physical activity programs [82].

Process models had the greatest diversity of functions and intentions based on the goals for the development and exploration stages of the project. Unlike process evaluations that examine the components of the intervention to determine what factors are leading to the desired outcomes [83], ﻿process models aim to provide a guide to translating existing knowledge into practice [21]. In some cases, researchers selected process models that fit their specific objectives [37, 60]. Reid et al. [39] utilized the integrated capacity-building framework as their focus was to identify and build collaborations that are necessary for the successful implementation of their obesity prevention interventions in different communities across New York State. Alternatively, Pallan et al. [51] employed the 46-item Typology of Cultural Adaptation and Programme Theory of Adapted Health Promotion Interventions Checklist to support the adaptation of an existing physical activity program for Bangladeshi and Pakistani families in Birmingham, United Kingdom. In contrast, process models can be all-encompassing, covering a variety of components. For example, the CBPM Framework involves the recruitment of organizations and members of the community, tailoring the program to meet the specific needs of the community, and describing how the program and participating organizations will be managed [72].

While aspects of determinant frameworks were incorporated into some of the process models and evaluation frameworks, only one framework was used to explore the factors that influence program implementation. As a result, it is difficult to make conclusions on the prominent determinant frameworks and components. Instead of using a framework, studies included an examination of barriers and enablers to implementation and program engagement through inductive focus groups with partners, service providers, parents, and/or children [84, 85]. Few studies have explored the factors that influence the dissemination of programs, such as feasibility, knowledge, motivation of providers, social context, and the structure of the service providers [14]. Future studies should consider utilizing determinant frameworks, such as the A + quality improvement toolkit, or alternative frameworks not identified in this review (e.g., Theoretical Domains Framework [86] or the Consolidated Framework for Implementation Research (CFIR) [87]) to guide the assessment of barriers and enablers that affect the adoption, dissemination, and effectiveness of community-based programs [88].

In terms of implementation evaluation, RE-AIM was the most frequently used framework. Using a socio-ecological approach, this framework utilizes individual-level (reach and efficacy) and organizational-level dimensions (adoption, implementation, and maintenance) to evaluate the implementation of population-level programs [13]. While all of the evaluation frameworks examine the effectiveness and implementation fidelity, RE-AIM is particularly beneficial for community-based programs since it considers the long-term benefits of the program by integrating the component of maintenance [13], which is missing from the Hybrid Type 3 Evaluation Design framework. As community-based programs are commonly critiqued for their ability to sustain long-term outcomes, supporting the maintenance of these interventions is essential [89]. Alternative evaluation frameworks should be tested for their applicability in examining the implementation of physical activity programs for children, such as the Exploration, Preparation, Implementation, Sustainment (EPIS) Framework [90] or the Practical, Robust Implementation and Sustainability Model (PRISM) [91].

While implementation models and frameworks tend to focus on the development, exploration, or evaluation of programs, they can be flexible and utilized in multiple situations. For instance, evaluation frameworks are designed to examine the effectiveness and quality of program implementation, but they can also offer questions to consider during the development and exploration of community-based physical activity programs. For example, the RE-AIM framework was used by ﻿Hingle et al. [48] and ﻿Gittelsohn et al. [44] to guide the recruitment and adoption of their respective programs in addition to their evaluation. Thus, some models and frameworks can be used in more than one stage of the program implementation process.

Tailoring was the most prominent component across all three model/framework types [36, 70, 72–78], emphasizing the importance of adapting the program to meet the unique social and geographic needs of a specific community. As a strategy for tailoring programs during the development stage, process models that also integrate a situational analysis are advantageous. It has been suggested that considering the needs of the intended target population and the setting can increase the chances of program adoption and result in high implementation quality, as this process helps researchers and program developers align the program with the community’s values and resources during program development [92]. Researchers and program developers can conduct environmental assessments and interviews to gain a greater understanding of the social and environmental context, with the findings supporting the tailoring of programs [72–74]. Community capacity and developing partnerships were also frequent components included in process models. Collaborations with local stakeholders and families not only provide context on the community’s needs, but can also offer resources that support the dissemination of community-based programs that successfully address health issues in the community [93]. Finally, to develop effective community-based physical activity programs, models and frameworks should identify the essential features in the program structure that must be met in order to address children’s physical activity levels, such as the activity options and recruitment strategies [69, 79].

When implementing future community-based physical activity programs, it is recommended that researchers and program developers utilize implementation models and frameworks to guide the development, exploration, and evaluation of their programs. During the development stage, process models that integrate the local context into the program design can help programs meet the needs of the community. Identifying critical program features is also important, which can be done through a review of the literature and collaborations with local stakeholders. Models and frameworks that implement these components include the Behaviour Change Wheel [79], ANGELO Framework [71], and the Life Needs Model [75]. Due to the limited number of determinant frameworks, further research is needed on the frameworks and models that explore the factors that influence the implementation of community-based physical activity interventions. In terms of evaluating the implementation of programs, all of the evaluation frameworks identified in this review focus on implementation fidelity and the effectiveness of the program. RE-AIM is a prominent evaluation framework that can provide context to the adoption, dissemination, effectiveness, and maintenance of community-based programs. The Hybrid Type 3 Evaluation Design [58] is also a beneficial framework for evaluating program implementation.

One challenge for researchers and program developers moving forward is selecting an appropriate approach for their program. In implementation science, there are over 100 theories, models and frameworks. As highlighted by Estabrooks [94], further testing of proposed strategies and sharing detailed experiences about the application of models and frameworks in physical activity programs will provide insight into the generalizable and contextual factors associated with different process models, determinant frameworks and evaluation frameworks, as well as the components that influence clinical and implementation outcomes. In addition to the variety of models or frameworks available, it can be difficult to select one approach as it is typically not all-encompassing and does not contain all of the factors of interest; however, using multiple approaches makes it difficult to determine what factors are attributed to implementation outcomes [33]. Language has also contributed to this issue due to the inconsistency of terminology used in the field. As the discipline has developed, there has yet to be a gold standard or globally accepted definition for the concepts in implementation science [95]; consequently, a variety of terms are used for similar concepts. For instance, the component *partner management* encompasses a variety of terms in the literature, such as partnership planning [64], formalized procedures [68], and mobilizing the community [66]. Due to the lack of common terminology, it can be difficult to find models and frameworks with the desired components, and it is challenging to synthesize and apply the lessons learned from the literature and previous program evaluations into practice [96, 97]. While this study provides a list of potential approaches that can be used, implementation scientists must continue to work towards consistent language and terminology, and further examine the use of implementation models and frameworks in community-based physical activity programs for children.

### Limitations

While this study contributes to the implementation science literature, there are limitations that need to be addressed. As this is a scoping review, a quality assessment of the included articles was not conducted. Also, a grey literature search was not undertaken; consequently, this review may have missed community-based physical activity programs that were not developed or evaluated by a research team. Additionally, the findings from this study may be more generalizable to urban settings. While the studies in rural and remote areas were included in the review, a majority of the studies were located in urban or undisclosed areas. Interventions conducted and/or evaluated by researchers tend to take place in metropolitan areas where universities and academic institutions are located; therefore, further evaluations are needed to determine the unique recommendations for rural communities. Finally, implementation models and frameworks may have been used by researchers and program developers, but may have been missed if they were not reported by the authors of the excluded articles or were part of a large-scale project where the search missed the article with the implementation model/framework information.

## Conclusion

Implementation models and frameworks are beneficial when implementing community-based physical activity programs as they provide a guide for developing, exploring, and evaluating the quality and effectiveness of programs [21]. By tailoring programs to the local context, developing strong partnerships with appropriate community stakeholders, and integrating the factors that are essential for health behaviour change, researchers and program developers can create effective and sustainable public health initiatives. Implementation models and frameworks grew in prevalence in the 1990s; however, it takes a considerable amount of time to adapt research into new programs [98]. This can explain why all studies included in this review have taken place in the last decade, demonstrating implementation models and frameworks are becoming more prominent in the delivery of community-based physical activity initiatives but the use of these approaches is still in its infancy and warrants further exploration. This will require further investment in implementation science research, as well as evaluation of implementation models and frameworks, including their ability to adapt health promotion strategies to community settings [18]. Future studies should examine implementation models and frameworks when developing community-based physical activity programs to identify the most appropriate implementation model/frameworks for a community context and to ensure tailored, evidence-driven programs are being executed appropriately by program staff.

### Supplementary Information

**Additional file 1: Table S1. **Preferred Reporting Items for Systematic reviews and Meta-Analyses extension for Scoping Reviews (PRISMA-ScR) Checklist.

**Additional file 2: Table S2. **Search Strategy to Identify Articles Investigating the Implementation of Community-Based Physical Activity Programs.

**Additional file 3**: **Table S3.** Study Details from the Included Articles.

## Data Availability

All data generated or analysed during this study are included in this published article and its supplementary information files.

## Abbreviations

MVPA: Moderate-to-Vigorous Physical Activity
PRISMA-ScR: Preferred Reporting Items for Systematic Reviews and Meta-Analyses Extension for Scoping Reviews Checklist
ANGELO: Analysis Grid for Environments Linked to Obesity
CBPM: Community-Based Prevention Marketing
KTA: Knowledge-to-Action
CFIR: Consolidated Framework for Implementation Research
EPIS: Exploration, Preparation, Implementation, Sustainment
PRISM: Practical, Robust Implementation and Sustainability Model
