# Supplementary material for: Implementation models and frameworks used to guide community-based physical activity programs for children: a scoping review
Source: BMC Public Health. 2023 Aug 23;23:1604. doi: 10.1186/s12889-023-16465-2 (PMC10463798; doi:10.1186/s12889-023-16465-2)
Supplement: Supplementary file 2 — Additional file 2: Table S2. Search Strategy to Identify Articles Investigating the Implementation of Community-Based Physical Activity Programs. [file 12889_2023_16465_MOESM2_ESM.pdf]

Table S2. Search Strategy to Identify Articles Investigating the Implementation of Community-Based Physical Activity Programs

Database	Search String	Results
CENTRAL<br>(via Cochrane)	child* OR adolescen* OR youth* OR juvenile* OR pediatric* OR paediatric* OR child/ OR adolescent/<br>AND<br>physical activit* OR exercis* OR sport* OR recreation* OR fitness OR energy expenditure* OR exercise/ OR physical fitness/ OR energy metabolism/<br>AND<br>(implement* OR disseminat*) adj3 (framework* OR frame-work* OR model* OR plan* OR evaluat*)<br>OR<br>implement* OR implementation science* OR disseminat* OR protocol* OR guide* OR process* OR evaluat* OR scale-up OR implementation science/ OR health plan implementation/ guideline adherence/ OR practice guideline/<br>AND<br>communit* OR community based OR community-based OR community-oriented OR community-focused OR community focused OR community approach OR population level OR population-level OR societ* OR delivery of health care OR community health planning	4,980
MEDLINE (Ovid)	(child* OR adolescen* OR youth* OR juvenile* OR pediatric* OR paediatric*).tw.kf. OR child/ OR school child/ OR adolescent/<br>AND<br>(physical activit* OR exercis* OR sport* OR recreation* OR fitness OR energy expenditure*).tw,kf. OR exercise/ OR physical activity/ OR energy expenditure/<br>AND<br>(implement* OR disseminat*) adj3 (framework* OR frame-work* OR model* OR plan* OR evaluat*).tw,kf.<br>OR<br>(implement* OR implementation science* OR disseminat* OR protocol* OR guide* OR process* OR evaluat* OR scale-up).tw,kf. OR implementation science/ OR protocol compliance/ OR practice guideline/<br>AND<br>(communit* OR community based OR community-based OR community-oriented OR community-focused OR community focused OR community approach OR population level OR population-level OR societ*).tw,kf. OR community program/	4,263
Embase (Ovid)	(child* OR adolescen* OR youth* OR juvenile* OR pediatric* OR paediatric*).tw.kf. OR child/ OR school child/ OR adolescent/<br>AND<br>(physical activit* OR exercis* OR sport* OR recreation* OR fitness OR energy expenditure*).tw,kf. OR exercise/ OR physical activity/ OR energy expenditure/<br>AND<br>(implement* OR disseminat*) adj3 (framework* OR frame-work* OR model* OR plan* OR evaluat*).tw,kf.<br>OR<br>(implement* OR implementation science* OR disseminat* OR protocol* OR guide* OR process* OR evaluat* OR scale-up).tw,kf. OR implementation science/ OR protocol compliance/ OR practice guideline/	6,638

	AND<br>(communit* OR community based OR community-based OR community-oriented OR community-focused OR community focused OR community approach OR population level OR population-level OR societ*).tw,kf. OR community program/	
Scopus	(TITLE-ABS-KEY (child* OR adolescen* OR youth* OR juvenile* OR pediatric* OR paediatric*))<br>AND<br>(TITLE-ABS-KEY ("physical activit*" OR exercis* OR sport* OR recreation* OR fitness OR "energy expenditure*"))<br>AND<br>(TITLE-ABS-KEY ((implement* OR disseminat*) PRE/3 (framework* OR frame-work* OR model* OR plan* OR evaluat*)))<br>OR<br>(TITLE-ABS-KEY (implement* OR "implementation science*" OR disseminat* OR protocol* OR guide* OR process* OR evaluat* OR scale-up))<br>AND<br>(TITLE-ABS-KEY ((communit* OR "community based" OR community-based OR community-oriented OR community-focused OR "community focused" OR "community approach" OR "population level" OR population-level OR societ*))	9,999
CINHAL	TI (child* OR adolescen* OR youth* OR juvenile* OR pediatric* OR paediatric*) OR AB (child* OR adolescen* OR youth* OR juvenile* OR pediatric* OR paediatric*)<br>AND<br>TI (physical activit* OR exercis* OR sport* OR recreation* OR fitness OR energy expenditure*) OR AB (physical activit* OR exercis* OR sport* OR recreation* OR fitness OR energy expenditure*)<br>AND<br>TI ((implement* OR disseminat*) N3 (framework* OR frame-work* OR model* OR plan* OR evaluat*)) OR AB ((implement* OR disseminat*) N3 (framework* OR frame-work* OR model* OR plan* OR evaluat*))<br>OR<br>TI (implement* OR implementation science* OR disseminat* OR protocol* OR guide* OR process* OR evaluat* OR scale-up)) OR AB (implement* OR implementation science* disseminat* OR protocol* OR guide* OR process* OR evaluat* OR scale-up)<br>AND<br>TI (communit* OR community based OR community-based OR community-oriented OR community-focused OR community focused OR community approach OR population level OR population-level OR societ*) OR AB (communit* OR community based OR community-based OR community-oriented OR community-focused OR community focused OR community approach OR population level OR population-level OR societ*)	3,468

Web of Science	TS=(child* OR adolescen* OR youth* OR juvenile* OR pediatric* OR paediatric*)<br>AND<br>TS=(physical activit* OR exercis* OR sport* OR recreation* OR fitness OR energy expenditure*)<br>AND<br>TS=((implement* OR disseminat*) NEAR/3 (framework* OR frame-work* OR model* OR plan* OR evaluat*))<br>OR<br>TS=(implement* OR implementation science* OR disseminat* OR protocol* OR guide* OR process* OR evaluat* OR scale-up)<br>AND<br>TS=(communit* OR community based OR community-based OR community-oriented OR community-focused OR community focused OR community approach OR population OR population level OR population-level OR societ*)	12,449
