# Supplementary material for: Implementation models and frameworks used to guide community-based physical activity programs for children: a scoping review
Source: BMC Public Health. 2023 Aug 23;23:1604. doi: 10.1186/s12889-023-16465-2 (PMC10463798; doi:10.1186/s12889-023-16465-2)
Supplement: Supplementary file 3 — Additional file 3: Table S3. Study Details from the Included Articles. [file 12889_2023_16465_MOESM3_ESM.pdf]

Table S3. Study Details from the Included Articles

Author(s)	Year	DOI	Study Design	Study Location	Setting	Intervention Name	Study Purpose	Participant Details	Implementation Framework(s)
Baillie CP, Galaviz KI, Emiry K, Bruner MW, Bruner BG, Lévesque L.	2017	https://doi.org/10.1007/s13142-016-0428-2	Systematic review	USA, Canada & Australia	Unknown	Summary of 10 interventions	To explore the degree to which physical activity interventions that promote indigenous youth development report on internal and external validity factors.	Indigenous youth <18 years	RE-AIM
Beets MW, Weaver RG, Turner-McGrievy G, Huberty J, Ward DS, Freedman DA, Saunders R, Pate RR, Beighle A, Hutto B, Moore JB.	2014	https://doi.org/10.1016/j.cct.2014.05.013	Study Protocol	USA	Unknown	South Carolina Afterschool Alliance afterschool programs	To evaluate the effectiveness of HEPA strategies and to detail information on the delivery of intervention.	Children aged 6-12 years	Strategies To Enhance Practice (STEPs)
Bottorff JL, Huisken A, Hopkins M, Nesmith C.	2020	https://doi.org/10.1186/s12889-020-09737-8	Cross-sectional	Canada	Unknown	Healthy Together (HT)	To conduct an evaluation of a real-world, scale-up phase of the project using the RE-AIM framework.	Children aged 7–12 years	RE-AIM
Bryant CA, Courtney AH, McDermott RJ, Alfonso ML, Baldwin JA, Nickelson J, McCormack Brown KR, DeBate RD, Phillips LM, Thompson Z, Zhu Y.	2010	https://doi.org/10.1111/j.1746-1561.2010.00493.x	Case Study	USA	Urban	VERB Summer Scorecard	To describe the design of the VERB program.	Youth aged 9-13 years	Community-Based Prevention Marketing (CBPM) Framework
Gipson CM, Campbell N, Malcom NL.	2018	https://doi.org/10.3390/sports6030100	Case Study	USA	Rural	CrossFit Kids	To detail the partnership implementation process undertaken to develop and deliver a Crossfit physical activity program for 'at-risk' youth in a low socioeconomic area.	At-risk youth (targets for negative stigmatization, discrimination, sanctioning and low self-perceptions) aged <18 years	Management Model for Sport and Physical Activity Community-based Partnerships
Gittelsohn J, Novotny R, Trude AC, Butel J, Mikkelsen BE.	2019	https://doi.org/10.3390/ijerph16010030	Case Study	USA	Remote	Children's Healthy Living (CHL) program	To describe the program's building community capacity and promoting sustainability training.	Children aged 2–8 years	ANGELO Framework<br>RE-AIM
Hill JL, Zoellner JM, You W, Brock DJ, Price B, Alexander RC, Frisard M, Brito F, Hou X, Estabrooks PA.	2019	https://doi.org/10.1186/s12889-019-6450-9	Quasi-experimental Trial	USA	Urban	iChoose intervention	To describe the steps taken to identify, develop and preliminary feasibility testing of a locally-relevant, childhood obesity treatment program for a community experiencing health disparities.	Children aged 8 to 12 years	RE-AIM
Hingle MD, Turner T, Kutob R, Merchant N, Roe DJ, Stump C, Going SB.	2015	https://doi.org/10.1186/s12889-015-2595-3	Study Protocol	USA	Urban	EPIC Kids	To describe the design and evaluation of the EPIC Kids Study.	Children aged 9 to 12 years	RE-AIM
Hughey SM, Weaver RG, Saunders R, Webster C, Beets MW.	2014	https://doi.org/10.1016/j.evalprogplan.2014.04.004	Quasi-experimental Trial	USA	Unknown	YMCA Afterschool Programs	To describe the process evaluation outcomes in after-school programs.	children ages 5–12 years	Systems Framework
Huhman M, Kelly RP, Edgar T.	2017	https://doi.org/10.1007/s13679-017-0252-0	Review	USA	Urban	VERB Summer Scorecard	To summarize the history of the VERB campaign's social marketing approach and its effectiveness in promoting behavior change.	Youth aged 9-13 years	Social Marketing Model
Jung ME, Bourne JE, Gainforth HL.	2018	https://doi.org/10.1186/s12966-017-0638-0	Case Study	Canada	Unknown	Healthy Together (HT)	To use the RE-AIM framework to comprehensively evaluate the first iteration of Healthy Together.	Children aged 7-18 years (module 2 tailored for children aged 7-12 years)	RE-AIM
Koorts H, Gillison F.	2015	https://doi.org/10.1186/s12889-015-2466-y	Case Study	UK	Unknown	Unknown	To using the RE-AIM framework to report on the project's implementation and to review the methodological challenges of conducting an evaluation of existing community-based program using RE-AIM.	Children and adolescents aged 7–14 years	RE-AIM
Liu S, Weismiller J, Strange K, Forster-Coull L, Bradbury J, Warshawski T, Naylor PJ.	2020	https://doi.org/10.1186/s12887-020-02297-1	Prospective Evaluation	Canada	Urban	Mind, Exercise, Nutrition … Do it! (MEND) childhood obesity intervention	To describe and explore program reach, attendance, satisfaction, acceptability, fidelity, facilitators and challenges to implementation during the scale-up of MEND B.C., and monitoring program effectiveness.	Children and adolescents aged 7–13 years	Hybrid Type 3 Evaluation Design
Mikkelsen BE, Novotny R, Gittelsohn J.	2016	https://doi.org/10.3390/ijerph13101023	Case Study	USA	Remote	Children's Healthy Living (CHL)	To proposes methodology, guidelines and directions for future research through analysis and examination strengths and weaknesses in the programs.	Children aged 2–8 year	RE-AIM
Moores CJ, Miller J, Perry RA, Chan LL, Daniels LA, Vidgen HA, Magarey AM.	2017	https://doi.org/10.1186/s12889-017-4907-2	Case Study	Australia	Unknown	Parenting, Eating and Activity for Child Health (PEACH™) program	To describe the translation of the evaluation framework to scale-up a randomised controlled trial (RCT) to a large-scale community intervention.	Families of overweight children	RE-AIM
Nigg C, Geller K, Adams P, Hamada M, Hwang P, Chung R.	2012	https://doi.org/10.1007/s13142-012-0120-0	Longitudinal Study	USA	unknown	The Fun 5 program	To present dissemination results from an evidence-based intervention.	Children in grades 4–6 (aged 8-12 years)	RE-AIM
O'Neil ME, Fragala-Pinkham M, Ideishi RI, Ideishi SK.	2012	https://doi.org/10.3109/01942638.2012.668089	Case Study	USA	Unknown	The Kids Fitness Program	To share their experiences designing, implementing, and evaluating health promotion programs in community settings.	School-aged children (6–13 years)	Life Needs Model

Pallan M, Griffin T, Hurley K, Lancashire E, Blissett J, Frew E, Gill P, Griffith L, Jolly K, McGee E, Parry J.	2019	https://doi.org/10.1186/s12889-019-7159-5	Study Protocol	UK	Urban	Child weigHt mANaGement for Ethnically diverse communities (CHANGE) study	To describe the cultural adaptations made to the First Steps program for Pakistani and Bangladeshi families.	children aged 4–11 years (primarily Pakistani and Bangladeshi families)	The Behaviour Change Wheel Typology of Cultural Adaptation and Programme Theory
Pallan M, Griffin T, Hurley KL, Lancashire E, Blissett J, Frew E, Griffith L, Hemming K, Jolly K, McGee E, Thompson JL.	2019	https://doi.org/10.3310/hta23330	Randomised Controlled Trial	UK	Urban	Child weigHt mANaGement for Ethnically diverse communities (CHANGE) study	To report the process of cultural adaptation of the First Steps program for Pakistani and Bangladeshi families.	Children aged 4–11 years	The Behaviour Change Wheel Typology of Cultural Adaptation and Programme Theory
Pallan M, Hurley KL, Griffin T, Lancashire E, Blissett J, Frew E, Gill P, Hemming K, Jackson L, Jolly K, McGee E.	2018	https://doi.org/10.1186/s40814-018-0373-6	Randomised Controlled Trial	UK	Urban	Child weigHt mANaGement for Ethnically diverse communities (CHANGE) study	To assess the acceptability of the programme to families attending and the feasibility of programme delivery to inform the design of a future randomised evaluation of the programme.	Children aged 4–11 years	The Behaviour Change Wheel
Parent MM, Harvey J.	2017	https://doi.org/10.1080/17430437.2015.1124561	Longitudinal study	Canada	Urban	Kids in Shape (KIS)	To assess the partnership component of a community-based youth sport for development programme.	Children aged 6–12 years	Parent and Harvey (2009) model
Reid KS, Sekhobo JP, Gantner LA, Holbrook MK, Allsopp M, Whalen LB, Koren-Roth A.	2018	https://doi.org/10.1016/j.evalprogplan.2017.12.004	Case study	USA	Urban, Suburban Rural	New York State Eat Well Play Hard–Community Projects (EWPH-CP)	To assess whether and how community-based coalitions funded under the New York State Eat Well Play Hard Initiative built capacity for policy, systems, and environmental (PSE) changes to prevent childhood obesity in their communities.	Dependent on program	Integrated Capacity Building Framework
Weaver RG, Beets MW, Hutto B, Saunders RP, Moore JB, Turner-McGrievy G, Huberty JL, Ward DS, Pate RR, Beighle A, Freedman D.	2015	https://doi.org/10.1093/her/cyv052	Randomized Controlled Trial	USA	Urban, Suburban, Rural	South Carolina Afterschool Alliance afterschool programs	To describe the implementation strategies based on STEPs for HE-PA, quality of implementation, and how the STEPs implementation was associated with the main outcomes.	Children aged 6-12 years	Strategies-To-Enhance-Practice (STEPs)
Wiecha JL, Hannon C, Meyer K.	2013	https://doi.org/10.1177/1524839912455644	Case study	USA	Urban	YMCA afterschool programs	To describe formative and pilot test results of A+, a QI toolkit for health promotion in afterschool programs.	Children ages 5–12 years	A+, a Quality Improvement (QI) Toolkit
Wilken LR, Novotny R, Fialkowski MK, Boushey CJ, Nigg C, Paulino Y, Guerrero RL, Bersamin A, Vargo D, Kim J, Deenik J.	2013	https://doi.org/10.1186/1471-2458-13-944	Study Protocol	USA	Remote	Children's Healthy Living (CHL) Program	To rationalize and design a community randomized environmental intervention trial and program evaluation.	Children ages 2-8 years who live in the Pacific region	RE-AIM
Willows N, Dyck Fehderau D, Raine KD.	2016	https://doi.org/10.1111/hsc.12229	Case study	Canada	Rural	Obesity prevention strategies	To implement an action-based workshop using the ANGELO framework to create community-relevant strategies to prevent childhood obesity.	Indigenous children aged <18 years	ANGELO Framework
Wurz A, Bean C, Shaikh M, Culos-Reed SN, Jung ME.	2021	https://doi.org/10.1111/hsc.13596	Case study	Canada	Urban	The Bounce Back League	To describe three cases of successful physical activity implementation among diverse populations.	Children aged 9-12 years from under-resourced communtities	Knowledge-To-Action (KTA) Framework
